# Polygonum Cuspidatum as a Laxative

**Published:** 1908-08-29

**Authors:** 


### THERAPEUTICS.

### POLYGONUM CUSPIDATUM AS A LAXATIVE.

There are not many natural laxatives which contain derivatives of oxy-methyl-anthraquinone, although synthetic substances derived from it are used as purgatives. Purgatin or purgatol, for example, is an oxyanthraquinone derivative—diacetate of anthrapurpurin; and exodin is a similar synthetic preparation equivalent to the hexamethylester of rufigallic acid. Experiments and analyses have been carried out in France by MM. A. Goris and L. Crété upon the rhizome of *Polygonum cuspidatum*, and they have found that it contains natural laxative principles not very dissimilar to those of the trade preparations. *Polygonum cuspidatum* is a common garden plant, and its rhizome contains at least two glucosides. One of these is unnamed as yet, whilst the other has been called polygonin. Both yield emodin upon hydrolysis, and, in addition to the emodin, polygonin yields a sugar, whilst the unnamed glucoside yields what is probably emodin-methyl-ether. The active principle is considered to be the emodin which can also be obtained from aloes and from cascara sagrada.

The glucosides occur in the cells of the pith of the medullary rays, and of the parenchyma of both bast and cortex. Analyses show that the percentages of emodin present in the various parts are as follows:—

| | per cent. |
|---|---|
| In the fresh cortex ... ... ... | 0.556 |
| ,, dry cortex ... ... ... | 1.200 |
| ,, fresh pith ... ... ... ... | 0.629 |
| ,, dry pith ... ... ... ... | 1.400 |
| ,, fresh entire rhizome ... ... | 0.353 |
| ,, dry entire rhizome ... ... | 0.676 |

The powdered rhizome has been used medicinally; the dose required is approximately double that of Chinese rhubarb, and the laxative result has been found to be excellent.

